# Pivotal Response Treatment with and without robot-assistance for children with autism: a randomized controlled trial

**DOI:** 10.1007/s00787-021-01804-8

**Published:** 2021-06-03

**Authors:** Iris van den Berk-Smeekens, Manon W. P. de Korte, Martine van Dongen-Boomsma, Iris J. Oosterling, Jenny C. den Boer, Emilia I. Barakova, Tino Lourens, Jeffrey C. Glennon, Wouter G. Staal, Jan K. Buitelaar

**Affiliations:** 1 Karakter Child and Adolescent Psychiatry University Centre, Reinier Postlaan 12, 6525 GC Nijmegen, The Netherlands; 2 Donders Institute for Brain, Cognition and Behavior, Radboud University Medical Centre, Nijmegen, The Netherlands; 3 Karakter Child and Adolescent Psychiatry, Ede, The Netherlands; 4 Department of Industrial Design, Technical University Eindhoven, Eindhoven, The Netherlands; 5 TiViPe, Helmond, The Netherlands; 6 Conway Institute of Biomolecular and Biomedical Research, School of Medicine, University College Dublin, Dublin, Ireland; 7 Institute for Brain and Cognition, Leiden University, Leiden, The Netherlands

**Keywords:** Autism spectrum disorder (ASD), Pivotal response treatment (PRT), Robotics, Randomized controlled trial (RCT)

## Abstract

Pivotal response treatment (PRT) is a promising intervention focused on improving social communication skills in children with autism spectrum disorder (ASD). Since robots potentially appeal to children with ASD and may contribute to their motivation for social interaction, this exploratory randomized controlled trial (RCT) was conducted comparing PRT (PRT and robot-assisted PRT) with treatment-as-usual (TAU). Seventy-three children (PRT: *n* = 25; PRT + robot: *n* = 25; TAU: *n* = 23) with ASD, aged 3–8 years were assessed at baseline, after 10 and 20 weeks of intervention, and at 3-month follow-up. There were no significant group differences on parent- and teacher-rated general social-communicative skills and blindly rated global functioning directly after treatment. However, at follow-up largest gains were observed in robot-assisted PRT compared to other groups. These results suggest that robot-assistance may contribute to intervention efficacy for children with ASD when using game scenarios for robot-child interaction during multiple sessions combined with motivational components of PRT. This trial is registered at https://www.trialregister.nl/trial/4487; NL4487/NTR4712 (2014-08-01).

**Supplementary Information:**

The online version contains supplementary material available at 10.1007/s00787-021-01804-8.

## Introduction

Autism spectrum disorder (ASD) is an early onset neurodevelopmental disorder characterized by deficits in social communication and interaction, and by restricted repetitive patterns of behavior, interests, or activities [1]. Many children with ASD face poor outcomes regarding social interaction and independent functioning [1]. Providing appropriate intervention approaches for young children with ASD is deemed important for obtaining optimal outcomes in later life [2, 3].

A systematic review identified 27 interventions for ASD that met the criteria for evidence-based practice, of which the majority for (young) children are based on techniques of applied behavior analysis (ABA) [4]. Pivotal Response Treatment (PRT) is a widely known naturalistic (transfer-focused) and feasible ABA-based behavioral intervention. In PRT, the focus is on "pivotal" (core) areas that, when targeted, can improve other untargeted areas of functioning and skills [5]. Pivotal areas include (1) motivation to engage in social communication, (2) responsiveness to multiple cues, (3) self-initiations and (4) self-management [6].

Implementation strategies of PRT include: following the child’s interests, gaining the child’s attention, using clear instructions (prompts), providing immediate and contingent reinforcement in response to a child’s initiation or good attempt, and interspersing maintenance and acquisition tasks. Family involvement in the treatment (i.e., parent training) and implementation in both home and school contexts are important aspects of PRT since it facilitates generalization of acquired skills in daily life [5].

So far, reviews of more than 50 (mostly non-randomized) PRT studies for children with ASD reported promising results on social communication and functioning and self-initiations specifically [7–9]. In the few randomized controlled trials (RCTs) available, high variability in dose ranging from 60/90 min a week during 12 weeks [10] to 600 min a week during 26 weeks [11] is reported. Results from these studies indicate that PRT for children with ASD may enhance the child’s number and mean length of functional utterances (both elicited and spontaneous) [10, 12, 13], general social-communication skills [10, 13], and global clinical functioning [10, 14]. Despite these promising results most of the currently conducted trials are limited by small sample sizes (*n* < 50), a lack of an intention-to-treat approach, and limited follow-up measures. In addition, a gap exists in studies investigating specific components or ways of delivery that may enhance PRT effectiveness, while identifying these intervention-enhancing components is key for achieving optimal outcomes for children with ASD [15, 16].

In search of effective components of interventions for children with ASD, the use of technology such as robots has received increasing interest during the last decade [17, 18]. In contrast to other technologies, robots provide embodied multi-modal features (speech combined with gestures and other movements) that are important when training social-communicative behavior. Robots are appealing to many children with ASD, which may contribute to more positive affect [19] and to a higher motivation for social interaction [20]. Additionally, children with ASD may show more attention towards a robot compared to a human trainer, which can positively affect their learning opportunities [21, 22]. Also, since children with ASD show higher intolerance of uncertainty [23], robots may be useful in showing higher behavioral predictability by simplified social uses and more repetition [24, 25]. The combination of the multimodal features and behavioral predictability of robotics combined with eliciting more motivation and attention in children with ASD can positively affect the learning opportunities within interventions for this target group. Since focusing on motivation for social interaction and gaining child’s attention are key elements of PRT, robot-assistance may further enhance this intervention model. However, robot-assistance in PRT has not yet been investigated. Single-case design studies on the use of other robot-assisted ABA-based interventions for children with ASD (using a small sample of *n* = 6) with a low dosage (e.g., six 15/20-min sessions or four 10-min sessions) suggested increased communication and social interaction after the intervention [26, 27] although no additional gains were found for a robot-assisted condition compared to a non-robot condition [26, 28].

In conclusion, well-designed RCT’s on PRT for young children are limited. Additional controlled trials are needed to examine the effects of PRT on targeted and untargeted areas of functioning and to explore the role of robot-assistance in this treatment model. Therefore, the present exploratory study was designed to examine the efficacy of PRT (with and without robot-assistance) compared to treatment-as-usual (TAU) in a clinical sample of young children with ASD (*n* = 73). The study included a randomized controlled design with an intention-to-treat approach, blinded and non-blinded outcome measures, and a follow-up assessment. The main objectives were to assess the efficacy of PRT (with and without robot-assistance) compared to TAU in (1) improving general social-communicative skills and (2) improving clinical global functioning. Secondary objectives were to examine treatment outcomes on ASD symptom severity, parenting stress, and self-initiations during PRT, and to explore the influence of child-, parent- and intervention-related factors on treatment effectiveness. We hypothesized treatment outcomes for all groups, with the largest improvement for children who received robot-assisted PRT, and also more improvements for children who received PRT, compared to TAU.

## Method

### Study design

This study involved an exploratory three-armed RCT and was conducted within the context of clinical outpatient units of Karakter, a tertiary, multi-site centre for complex child and adolescent psychiatry in The Netherlands.

Seven different outpatient sites of Karakter were involved in this study, all using the same procedures and clinical protocols. We utilized an adaptive design to measure the effects of clinical outpatient treatment of children with ASD in this naturalistic sample. Participants were randomly assigned to either PRT, robot-assisted PRT or TAU (1:1:1). Stratification was conducted based on age, total intelligence quotient (TIQ), and site, since treatment outcomes in young children with ASD may vary by age [29, 30] and TIQ [30]. Participants were matched manually on these variables by an investigator not involved in data collection and outcome assessment. The protocol of the study was registered in the Netherlands Trial Register (NL4487/NTR4712, https://www.trialregister.nl/trial/4487, contact author for full trial protocol). The study was approved by the Local Ethics Committee (CMO Arnhem-Nijmegen, NL50509.091.14) and all procedures were in accordance with the 1964 Helsinki declaration and its later amendments.

### Participants

Participants were included when they met the following inclusion criteria; (1) a primary clinical diagnosis of ASD, according to the DSM-IV classification; (2) aged 3–8 years; 3) a TIQ of 70 or higher; (4) ability to speak with one-word utterances at minimum, and (5) at least one of the parents speaks Dutch to the child. An exclusion criterion was having received PRT previously to eliminate the impact of earlier intervention. Within our sample of children with complex psychiatric problems, comorbid psychiatry disorders were allowed, but the primary diagnosis (and intervention) of the child had to be focused on ASD. For participants that were assigned to one of the PRT groups dosages of medication must be kept stable before start of the intervention, but not for the TAU group since pharmacotherapy is part of regular care for this complex target group. However, participants were not excluded if dosages changed due to the intention-to-treat (ITT) approach.

In the trial protocol, adherence to the ASD cut-off on the Autism Diagnostic Observation Schedule-Second edition (ADOS-2) [31] was defined as an eligibility criterion. Of participants that were included in the analyses, 89% scored above the ASD cut-off. In the remaining 11%, participants scored one point (*n* = 4) or two points (*n* = 3) below the cut-off, or the ADOS-2 could not be fully administered due to behavior problems that severely interfered with administration (*n* = 1). These participants were included in the study as they had a very clear clinical ASD diagnosis, based on a thorough multidisciplinary and multi-informant psychiatric examination. Children with an ADOS-2 score below cut-off were equally distributed to treatment groups by randomization (*X*^2^(2) = 0.24, *p* = 0.886).

### Procedures

Participants were recruited from clinical outpatient referrals to Karakter. The authoritative caregivers (further called ‘parents’) of the participants received verbal and extensive written information on the outline and aims of the study and signed an informed consent form prior to inclusion. After screening for study eligibility, the participants were randomly assigned to a group. Prior to enrolment and baseline measures, parents received psycho-education on ASD if not received in the past. PRT and robot-assisted PRT consisted of 20 sessions of therapy, once a week, by certified PRT-therapists who were trained to reach a fidelity score of over 80% (level III). In total, 13 certified PRT-therapists were involved in this study. Meetings with a certified PRT-trainer (level V) were held for additional training and supervision. For participants that were randomly assigned to TAU, treatment was indicated by a clinician based on ‘shared decision-making’ with parents.

### PRT protocol

The PRT and robot-assisted PRT protocols were based on the Dutch translation of the PRT manual [32], focusing on implementing three-term contingency learning trials in social communicative skills: (1) presenting a clear opportunity (incorporating the child’s choice and gaining the child’s attention), (2) target behavior (child’s initiation or prompting the child to initiate), (3) reinforcing the child’s initiation or attempt naturally and contingently. Target behavior was in both the PRT and robot-assisted PRT group adjusted to the child’s current level of social communication (e.g., two (or more)-utterances, asking for an object/activity, asking for help, protesting, wh-questions, responding to multiple cues). In treatment, the focus was mainly on teaching parents to implement PRT principles in the natural environment of the child. Further, teachers were involved in implementing PRT techniques at school. Both PRT and robot-assisted PRT consisted of 14 parent–child sessions, 4 parent-only sessions, and 2 teacher sessions. Each PRT session had a duration of 45 min, except for one teacher session including a 90-min school/day-care visit.

#### PRT

In the parent–child sessions, the therapist modelled the PRT techniques during therapist-child interaction, after which parents practiced the PRT techniques during parent–child interaction while being coached by the therapist. Dependent on parent and child characteristics and target goals, the focus was on specific PRT techniques. These sessions were recorded on tape for later analysis. In the parent-only sessions, the progress of the child on individual target behaviors was discussed as well as the parental use of the PRT techniques at home. In the teacher sessions, the child's teacher was involved in discussing and practicing the use of the PRT techniques at school.

#### Robot-assisted PRT

In the robot-assisted PRT, a NAO robot was added in the first 15 min of each of the parent–child sessions (but not in the teacher sessions). Target behaviors were practiced during robot-child interactions, in which the robot was controlled by the PRT therapist. Motivational techniques of PRT were incorporated into game scenarios for robot-child interaction (see [33] for a description of the development and protocol) using the three-step contingency: (1) therapist controlled the robot in providing antecedent stimulus, (2) therapist controlled the robot in providing prompt(s) for child’s target behavior, (3) therapist controlled the robot in reinforcing the (attempt to) target behavior naturally and contingently. Supplementary Information 1 shows details on how the robot behavior was controlled and on the game scenarios for robot-child interaction. Parents were asked to observe how PRT techniques were used and the therapist modelled the techniques to the parent by implementing learning opportunities by use of the robot. After the robot-assisted part of the session (i.e., 15 min), the session was continued as similar to the PRT condition: parents practiced the PRT techniques during parent–child interaction and were coached by the therapist. The parent-only sessions and teacher sessions were also similar to the PRT condition.

#### Fidelity of PRT implementation

Parental Fidelity of PRT Implementation at the end of the intervention was assessed using the partial interval recording procedure as described by Verschuur et al. [34]. In this procedure, the fidelity is based on a sequence of correctly implemented PRT components that constitute a three-term contingency, instead of scoring each PRT component separately. In the current study, 10-min video probes of the last two recorded parent–child sessions were used and a mean of these two was calculated to determine the total percentage of fidelity for each parent. Videos were coded by a trained research assistant, blinded to group assignment. Of the video probes, 20% was coded by a naïve second rater resulting in excellent agreement (intraclass correlation coefficient = 0.97).

#### Treatment-as-usual (TAU)

The TAU condition consisted of guidance of parents, intensive family therapy, treatment at school (e.g., mediation), social skill training groups, pharmacotherapy, or a combination of these. These patient/family-tailored treatments ranged in intensity and frequency (from 1.5 h per week to 1 h per month). After an intervention period of 20 weeks, all participants in the TAU group were offered the possibility to receive PRT (of which five actually received PRT).

### Measures

#### Demographic information

Demographics on participant characteristics (i.e., age, gender, psychiatric comorbidity, and medication use) and parental characteristics (i.e., presence of psychopathology, education level) were extracted from case files and intake questionnaires of Karakter. TIQ of the child was estimated by either the Wechsler Intelligence Scale for Children (WISC-III) [35], Wechsler Preschool and Primary Scale of Intelligence (WPPSI-III) [36], or Mullen Scales of Early Learning (MSEL) [37]. If TIQ could not be estimated, approximate IQ was determined based on the child’s mental age and/or educational performance.

#### Primary outcomes

General social-communicative skills in the child’s natural environment were assessed using the Social Responsiveness Scale, preschool and child version (SRS) [38, 39]. The 65-item digitalized questionnaires, rated on a 4-point scale, were completed by the parent and teacher/daycare attendant at all time points: baseline, intermediate assessment point (week 10), endpoint (week 20), and follow-up (week 32).

Total raw scores were computed (based on the following subscales; Social Awareness, Social Cognition, Social Communication, Social Motivation, and Restricted Interests and Repetitive Behavior), with higher scores representing lower general social-communicative skills. The change score compared to baseline was used as a continuous outcome measure and the percentage of clinical responders, defined as a reduction of > 25% in total SRS scores compared to baseline was used as a categorical outcome.

Change in clinical global functioning was assessed at week 10, endpoint, and follow-up using the Clinical Global Impression-Improvement (CGI-I) [40], rated on a 7-point scale (*very much improved – score 1—to very much worse – score 7*) by experienced child- and adolescent psychiatrists who were unfamiliar with the participants and who were blinded to treatment allocation. Ratings were based on information about the clinical status of functioning, symptoms, and well-being in major areas of the participants life (i.e., home, school, relations). This information was provided by the coordinating therapist of the participant, who was instructed not to provide details on their group assignment or treatment phase. A clinical responder was defined as being *much improved* or *very much improved* on the CGI-I.

#### Secondary outcomes

The severity of ASD-related symptoms was assessed at baseline and endpoint with the Dutch version of the ADOS-2 [31]. The ADOS-2 was administered by a certified clinician who was blinded to treatment allocation and baseline outcomes. Of the calibrated severity score category (i.e., low: 1–4; moderate: 5–7; high: 8–10) based on Gotham et al. [41], a change score (endpoint-baseline) was computed. ADOS-2 Modules 1, 2, and 3 were used in 4.2%, 16.4%, and 78.1% of participants respectively at baseline (with 1.4% missing), and in 1.4%, 11.0%, and 74.0% of participants respectively at endpoint (with 13.7% missing). For 83.9% of participants, the same ADOS-2 module was used at baseline and endpoint.

Parenting stress was measured at all time points by the digitalized 34-items Dutch “Opvoedingsbelasting vragenlijst” (OBVL) [42]. A reliable clinical change on the OBVL was defined as a reduction of ≥ 4.03 points on the total *T*-score [42].

Specifically for both PRT groups, percentages of spontaneous appropriate initiations of the child were calculated at all time points during a semi-structured therapist-child interaction based on PRT guidelines [43]. A percentage was calculated dividing the number of therapist-elicited opportunities in which the child showed appropriate initiations without provided prompts (i.e., one-word sentences, two-word sentences, asking for object/activity, asking for help, wh-question asking (e.g., what, where, which, when), protesting, interrogating, making statements and responding to multiple cues) by the total number of protocolized, therapist-elicited opportunities.

### Statistical analyses

Since this present exploratory study was the first in estimating the effect of robot-assisted PRT and no research was available on comparing effects on the SRS and CGI resulting from either PRT or TAU, no power analysis for this three-group RCT could be performed. Measurements were continued after possible early termination of the intervention in line with the adaptive study design and intention-to-treat (ITT) analyses.

The ITT statistical analyses were conducted in accordance with the study protocol (two-tailed, *α* = 0.05). To compare the effect of PRT in general, analyses on primary and secondary outcomes were conducted comparing the two groups (PRT total versus TAU) and the three groups separately. Chi-square statistics were conducted for clinical responder analyses (SRS, CGI-I, ADOS, and OBVL). Sensitivity analyses were conducted with different cut-offs for clinical responders on the SRS. To examine group differences in SRS scores, analysis of variance (ANOVA) F-tests were computed with estimated change scores (baseline-endpoint and baseline-follow-up). Additionally, to compare percentages of spontaneous initiations at endpoint and follow-up between both PRT groups, independent sample *t*-tests were conducted. For each primary outcome, Bonferroni-holm corrections were implemented to account for multiple testing per reporter (i.e., parent, teacher or blinded clinician) on each time point (i.e., accounting two- versus three-group comparisons and use of both clinical responder measure and change scores for the SRS)[44]. Exploratory paired sample t-test (or Wilcoxon signed ranks test when the assumption of normality was violated) were conducted for within-group comparisons over time. All analyses were conducted using IBM SPSS Statistics version 25 [45]. Additional per-protocol analyses on the primary outcomes were conducted for participants that adhered ≥ 75% to the treatment protocol. To identify predictors of treatment response, clinical responders and non-responders were compared on child-, parent-, and intervention-related factors that were described in Table 1.

**Table 1:** Baseline Descriptive Characteristics and Hours of Treatment

	Mean (SD)/ *N* (%)			*F*(df), *X*^*2*^(df)	*p*
	PRT	PRT + robot	TAU		
	(*n* = 25)	(*n* = 25)	(*n* = 23)		
Age in years	6.43 (1.71)	6.18 (1.31)	6.09 (1.30)		0.704
Gender				0.61 (2)	0.739
Male	22 (88.00)	20 (80.00)	19 (82.61)		
Female	3 (12.00)	5 (20.00)	4 (17.39)		
TIQ	105.83 (15.18)	101.78 (14.18)	99.74 (13.24)	1.12 (2,67)	0.333
CGI- Severity	4.64 (1.22)	4.80 (0.82)	4.52 (0.89)	0.59 (2,69)	0.560
Psychiatric comorbidity^a^	10 (40.00)	7 (28.00)	12 (52.20)	1.46 (2,72)	0.232
AD(H)D	6 (24.00)	3 (12.00)	5 (21.70)		
AD(H)D + other	2 (8.00)	0 (0.00)	2 (8.70)		
Other	2 (8.00)	4 (16.00)	5 (21.70)		
Medication use^a^	6 (24.00)	6 (24.00)	6 (26.09)	0.04 (2)	0.982
Stimulants	5 (20.00)	3 (12.00)	2 (8.70)		
Stimulants + antipsychotics	1 (4.00)	0 (0.00)	3 (13.00)		
Antipsychotics	0 (0.00)	2 (8.00)	1 (4.30)		
Other	0 (0.00)	1 (4.00)	0 (0.00)		
Psychopathology mother^a^	8 (32.00)	8 (32.00)	8 (34.80)	0.06 (2)	0.973
Psychopathology father^a^	3 (12.50)	6 (24.00)	6 (27.30)	1.69 (2)	0.429
Education mother				3.17 (4)	0.530
Low	4 (16.70)	6 (24.00)	5 (21.70)		
Average	12 (50.00)	9 (36.00)	6 (26.10)		
High	8 (33.30)	10 (40.0)	12 (52.20)		
Education father				3.27 (4)	0.514
Low	5 (22.70)	6 (27.30)	7 (31.80)		
Average	10 (45.50)	5 (22.70)	8 (26.40)		
High	7 (28.00)	11 (50.00)	7 (31.80)		
Hours of treatment (week 0–20)	14.68 (4.92)	15.96 (6.20)	17.39 (9.30)	0.78 (2,70)	0.462

*AD(H)D* Attention-deficit (hyperactivity) disorder, *CGI* Clinical Global Impression, *F* test statistic resulting from analysis of variance, *N* number of participants, *p* *p* value (two-tailed), *PRT* group of participants who received Pivotal Response Treatment, *PRT* + *robot* group of participants who received robot-assisted Pivotal Response Treatment, *SD* standard deviation, *TAU* group of participants who received treatment-as-usual, *TIQ* total intelligence quotient, *X*^*2*^ test statistic resulting from chi-square analysis

Education level: low = primary or secondary education, average = intermediate vocational education, high = higher professional education/university

^a^Number of presence and percentage

## Results

### Study population

**Fig. 1:**
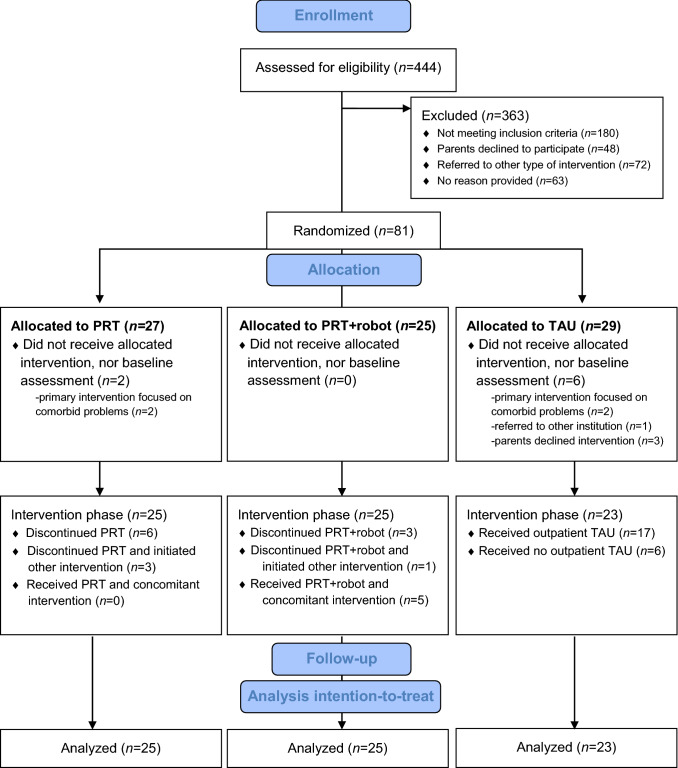
Consort participant flow diagram

Figure 1 shows the participant flow throughout the study. Eighty-one participants, recruited from June 2015 until December 2016 at seven different locations of Karakter, were randomized to one of the three arms. Of these, eight participants did not start with the allocated treatment, nor received baseline assessment, because the primary intervention focused on comorbid problems (*n* = 4), referral to another institution (*n* = 1), or parents declining intervention (*n* = 3). This resulted in 73 participants that initiated the treatment to which they were assigned. Non-adherence in the PRT intervention groups was related to comorbid psychiatric or family-related problems, which required additional interventions for some participants. Specifically, some participants either (1) discontinued PRT with no initiation of other intervention (PRT; *n* = 6, PRT + robot; *n* = 3), (2) discontinued PRT and initiated other intervention (PRT; *n* = 3, PRT + robot; *n* = 1), or (3) continued PRT with a concomitant intervention (PRT; *n* = 0, PRT + robot; *n* = 5). This resulted in 32 participants in the PRT total group (PRT; *n* = 16, PRT + robot; *n* = 16) that were included in the per-protocol analyses. In the TAU group, 17 participants received TAU, and 6 participants received no additional treatment at Karakter after psycho-education, resulting in 17 TAU participants in the per-protocol analyses. Missing values were equally distributed across groups and not imputed.

Baseline descriptive characteristics and hours of treatment did not differ between the groups (see Table 1). Change of medication dosages during the intervention occurred in all groups and was equally distributed (16% PRT + robot, 12% PRT, 13% TAU, respectively, *p* = 0.91). In both PRT groups, none of the parents showed adherence to the 80% PRT Fidelity of Implementation criterion at the end of treatment. The mean fidelity percentage for the PRT + robot group was 20% (SD = 15%) and for the PRT group 36%, (SD = 28%), *p* = 0.04.

### Primary outcomes

#### SRS continuous change score rated by parents and teachers

##### PRT total vs TAU

Change scores on the SRS rated by parents and teachers did not differ significantly between the PRT total group and the TAU group from baseline to endpoint (parents; *F*(1,69) = 1.14, *p* = 0.289, *d* = 0.28, teachers; *F*(1,65) = 0.30, *p* = 0.585, *d* = 0.13) and from baseline to follow-up (parents; *F*(1,68) = 2.02, *p* = 0.160, *d* = 0.41 teachers; *F*(1,65) = 0.19, p = 0.663, *d* = 0.11). There were no differences in change in general social-communicative skills between the total PRT group and TAU.

##### PRT vs PRT + robot vs TAU

**Fig. 2:**
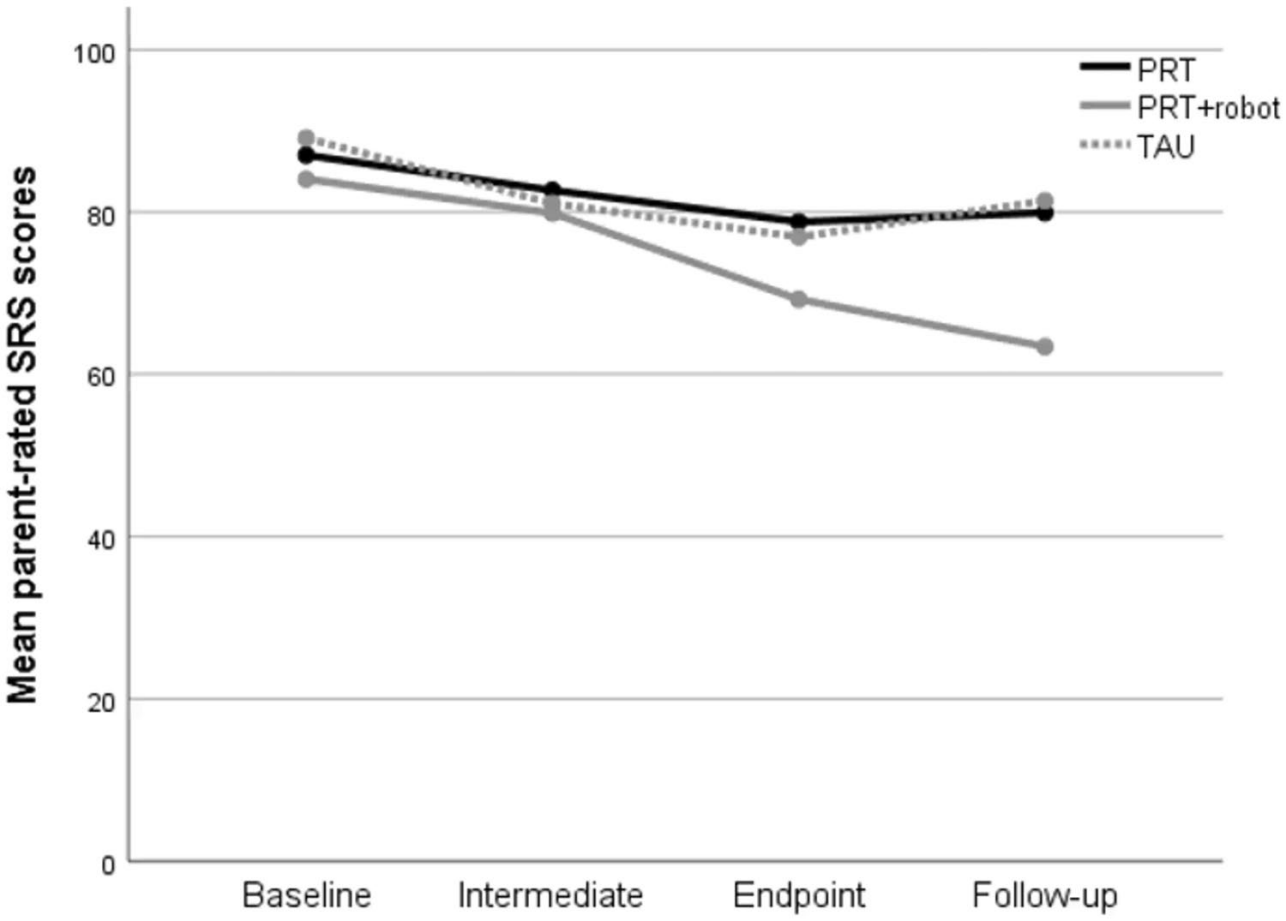
Change in total SRS scores rated by parents in PRT and PRT + robot and TAU from baseline to follow-up.

No significant differences were found between the three groups in the change scores from baseline to endpoint on the SRS rated by parents (*F*(2,69) = 2.55, *p* = 0.086). However, there were significant group differences in the continuous change scores from baseline to follow-up (*F*(2,68) = 6.67, *p* = 0.002, see Fig. 2). Post hoc analyses indicated a larger improvement in general social-communicative skills at follow-up for the PRT + robot group compared to the PRT group (*F*(1,48) = 9.38, p = 0.004, *d* = 0.88) and also compared to the TAU group (*F*(1,43) = 7.91, *p* = 0.007, *d* = 0.87). For the SRS rated by teachers, there were no differences between the three groups on SRS change scores (both baseline-endpoint and baseline-follow-up p > 0.05).

### SRS clinical responder rated by parents and teachers

#### PRT total vs TAU

Percentages of clinical responders on the SRS rated by parents and teachers did not differ significantly between the PRT total group and the TAU group at endpoint and follow-up (see Table 2).

**Table 2:** Percentages of clinical responders per group and results of chi-square analyses

	PRT total				TAU		Chi-square analyses results			
	% Endpoint		% Follow-up		% Endpoint	% Follow-up	Endpoint		Follow-up	
	PRT	PRT + robot	PRT	PRT + robot			*X*^2^(*df*)	*p*	*X*^2^(*df*)	*p*
Outcome										
Comparison										
SRS parents										
2-group	20.8		30.6		18.2	20.0	0.06 (1)	0.797	0.80 (1)	0.371
3-group	13.0	28.0	12.0	50.0			10.72 (2)	0.419	90.66 (2)	0.008**
SRS teachers										
2-group	27.3		25.6		18.2	21.7	0.66 (1)	0.417	0.12 (1)	0.729
3-group	13.0	42.9	16.7	36.8			50.75 (2)	0.050	20.47 (2)	0.291
CGI-I										
2-group	55.1		59.2		36.4	45.5	2.13 (1)	0.144	10.16 (1)	0.282
3-group	44.0	66.7	48.0	70.8			40.65 (2)	0.098	30.74 (2)	0.155
ADOS-2										
2-group	38.6				22.2		1.54 (1)	0.215		
3-group	13.6	63.6					130.81 (2)	0.009**		
OBVL										
2-group	64.6		63.3		66.7	57.9	0.23 (1)	0.867	0.17 (1)	0.683
3-group	73.9	56.0	64.0	62.5			10.72 (2)	0.423	0.18 (2)	0.914

*ADOS* Autism Diagnostic Observation Schedule second edition, *CGI-I* Clinical Global Impression- Improvement scale, *df* degrees of freedom, *OBVL* Dutch Opvoedingsbelasting vragenlijst, *p* *p* value (two-tailed); *PRT* = group of participants who received Pivotal Response Treatment, *PRT* + *robot* group of participants who received robot-assisted Pivotal Response Treatment, *PRT total* total group of participants that received PRT, *SRS* Social Responsiveness Scale, *TAU* group of participants who received treatment-as-usual, *X*^2^ test statistic resulting from Chi squared analyses, *2-group* comparison between PRT total and TAU, *3-group* comparison between PRT, PRT + robot and TAU

*p < .05, **p < .01

#### PRT vs PRT + robot vs TAU

No significant differences were found between the three groups in the percentage of clinical responders on the SRS rated by parents at endpoint. However, a higher percentage of clinical responders was found for the PRT + robot group compared with the PRT group and TAU group at follow-up (*χ*^2^(2) = 9.66, *p* = 0.008). For the SRS rated by teachers, there were no differences between the groups on the percentages of clinical responders at endpoint and follow-up, although the difference at endpoint showed a marginal trend towards significance, with a higher percentage in the PRT + robot group compared with the other groups (*χ*^2^(2) = 5.75, *p* = 0.050). Sensitivity analyses using Chi-square with other cut-off percentages for clinical response on the SRS confirmed the higher percentage of clinical responders in the PRT + robot group at follow-up (see Supplementary Information 2).

### CGI-I rated by blinded clinician

#### PRT total vs TAU

There were no significant differences between the groups on the percentage of participants that showed a clinically significant response on the CGI-I at endpoint and follow-up (see Table 2).

##### PRT vs PRT + robot vs TAU

As in the two-group comparison, there were no significant differences between the three groups on the percentage of clinical responders on the CGI-I at any time point (see Table 2).

### Secondary outcomes

#### ADOS-2 rated by blinded clinician

##### PRT total vs TAU

No significant differences in percentages of participants that showed a decrease in ADOS-2 severity category were found between PRT total group and the TAU group (see Table 2).

##### PRT vs PRT + robot vs TAU

A significant difference was found when comparing the three groups (*χ*^2^(2) = 9.66, *p* = 0.008); more children in the PRT + robot group showed a decrease in ADOS-2 severity category compared with both the PRT and the TAU group (see Table 2). No differences were found between the PRT group and the TAU group.

### OBVL rated by parents

#### PRT total vs TAU

No significant differences in percentages of participants that showed reliable clinical change on the OBVL at endpoint and follow-up were found between PRT total group and the TAU group (see Table 2).

#### PRT vs PRT + robot vs TAU

Although both the PRT + robot group and TAU group showed a decrease from baseline to follow-up on the paired sample *t*-tests (see Supplementary Information 3), there were no significant differences between the three groups (see Table 2).

### Spontaneous initiations

Although both PRT groups improved in percentages spontaneous appropriate initiations (see Supplementary Information 3), there were no significant differences between the groups at endpoint (*t*(36) = 1.05, *p* = 0.299) and follow-up (*t*(35) = − 1.02, *p* = 0.315).

Exploratory within-group analyses using paired-sample *t*-tests on the primary and secondary outcomes are described in Supplementary Information 3.

### Child-, parent- and intervention-related factors

A significantly higher proportion of females (45.5%) versus males (15.3%) were clinical responders on the SRS rated by parents at endpoint in the total sample (*X*^2^(1) = 5.29, *p* = 0.036). This effect was specifically found for the PRT group (*X*^2^(1) = 8.75, *p* = 0.034). Also, maternal education level marginally modified the percentages of clinical responders on the CGI-I at follow-up (*X*^2^(2) = 6.01, *p* = 0.050), with children of mothers with higher and moderate education levels showing higher gains. There were no effects of age, TIQ, ADOS severity, psychiatric comorbidity, medication use, psychopathology of parents, educational level of fathers, and amount of treatment hours on the SRS and CGI-I outcomes.

### Per-protocol analyses

After corrections for multiple testing, per-protocol analyses on the primary outcome measurements indicated a similar pattern of results as the ITT analyses, namely a larger improvement in general social-communicative skills at follow-up for the PRT + robot group compared to other groups.

## Discussion

This study is the first exploratory RCT of clinician- and parent-delivered PRT with and without robot-assistance compared to TAU in a clinical sample of young children with ASD. Strong points include blinded outcome measures and the inclusion of a follow-up assessment. As hypothesized, positive treatment outcomes in terms of improved general social-communicative skills were found for all groups, with the largest improvements for children who received robot-assisted PRT. However, the PRT total group and the TAU group did not differ in change of percentage of clinical responders in general social-communicative skills. This finding contradicts earlier RCTs that found larger gains for children receiving PRT compared to controls [10–13]. However, our treatment-as-usual condition included intensive patient- and family-tailored treatment for ASD focused on a variety of individualized target behaviors. This is in contrast to prior RCTs that used a standardized psycho-education group intervention besides community-based treatments [10], a structured one-to-one, clinician-led ABA focused on few target behaviors [13, 46], or a waiting list control group [11, 12] as a comparison.

The higher gains in the robot-assisted PRT group on the parent-rated SRS and the blindly-rated ADOS-2 suggest that robot-assistance may contribute to treatment efficacy for children with ASD when combined with motivational components of PRT, such as incorporating child-preferred activities, stimulus variation, direct-response-reinforcement relationships, and reinforcement of attempts. Robot-assistance may enhance positive learning opportunities by contributing to higher motivation and attention in children with ASD. Also, the multi-modal features of robotics may be useful when training social-communicative behavior while more consistent behavioral predictability may contribute to an attractive learning environment for children with ASD. However, the mechanisms underlying these treatment enriching effects of robot-assistance need further exploration.

Our findings are in contrast with other studies that used robotics in ABA-based interventions for children with ASD, who found no additional gains of robot-assistance [26, 28]. This may be explained by differences in design. In these previous studies, the behavioral repertoire of the robot was limited by a low differentiation of prompt levels, a technical assistant controlling the robot [28], very small samples (*n* = 3 to 6) and the robot-assistance being implemented in only four or five sessions. In contrast, in our study, a variety of prompt levels was used in a larger library of game scenarios, the robot was controlled by the PRT therapist and was implemented in a much larger group of children during a 20-week intervention. This suggests that implementing robot-assistance in more sessions using game scenarios that can be easily amended in complexity may enhance established interventions for children with ASD.

Our results highlight the contribution of parent-related factors since the largest gains on clinical global functioning were found in children of mothers with a moderate or higher education level. However, it is unclear whether this is due to further implementation of PRT techniques at home after the treatment was finished or a combination with other interventions since a naturalistic follow-up was used.

While parents reported increases in social-communicative skills, no increases were found in teacher ratings. Low correspondence between parent and teacher ratings of ASD-related symptoms has been reported earlier [47]. Possibly, the sensitivity of teacher ratings was limited and the implementation at school was rather an introduction to PRT (i.e., teachers were included in only two sessions). Future studies should focus on how to better embed PRT implementation at school.

In our study, exploratory results suggested a higher percentage of girls that showed clinical improvement in social-communicative skills compared to boys, while no effects were found for other child-related factors. Other PRT studies found high non-verbal problem-solving skills [10], or high expressive language skills, positive affect, appropriate toy contact, low social avoidance, and low stereotyped vocalizations [48] as predictors of treatment response. These findings warrant further study to investigate which child-related factors serve as robust modifiers on outcomes of PRT.

This study included several limitations. Although the results of the per-protocol analyses were similar to those of the intention-to-treat analyses and treatment intensity was not related to outcomes, it is notable that in both PRT groups participants discontinued the intervention or started a concomitant intervention. Due to the adaptive nature of our study design, we measured the naturalistic flow of treatment in this clinical outpatient group. While the PRT protocol was primarily focused on social communication goals, treatment for other ASD-related, comorbid, or family problems was required for many families as well (based on thorough multidisciplinary deliberation during the study). This study included children with complex psychiatric problems as is represented by (1) the comorbidity rates (around 40%) while earlier PRT trials did not include children with comorbidities [10, 11, 13, 14, 46] and (2) high rates of required psychopharmacological intervention use in this relatively young age group of children with ASD. Although standardized protocols are viewed as a strength of the PRT approach [8], integrating patient- and family tailored co-interventions may be necessary for many children with ASD and their families due to the complexity and heterogeneity of the condition. The similar results in the intention-to-treat analyses compared with the per protocol analyses suggests that integrating co-interventions with PRT is as effective as utilizing standardized protocols with no co-interventions. Furthermore, since we did not find lower parenting stress after highly protocolized PRT (but did find lower parenting stress at follow-up in the robot-assisted PRT and TAU groups), it may also be important to study how integration of specialized family-tailored interventions may contribute to lower stress and higher well-being. Although not necessarily a limitation, the contrasts between the health care system in The Netherlands and the United States of America and Canada contributed to differences in intensity and delivery of the current intervention (low intensity and outpatient orientated) compared to previous PRT trials that have been primarily conducted in Northern America. Currently, evidence is growing for a lower PRT intensity model that is more congruent with logistics of family routines and educational attendance while stimulating parents to use the PRT techniques also in between therapy sessions [11]. The results of the current study also support that a relatively low-intensity intervention (20 weeks) may be sufficient to improve the general social-communicative skills of children with ASD. However, since significant differences between groups were demonstrated at follow-up and not at earlier previous timepoints, further research is needed to get insight into the optimal dose and treatment delivery.

Also, it is notable that none of the parents in our study achieved the 80% Fidelity of Implementation criterion after the intervention. This may be due to (1) a high emphasis on parent implementation of PRT techniques during daily routines at home that were not recorded on video, rather than intensively during a video-recorded 10-min parent–child interaction and (2) the use of a different (and more stringent) fidelity coding system in this study, in which a correct sequence of PRT skills are highly emphasized, compared to fidelity measures with a more global calculation (scoring each PRT component separately) that are generally used [33]. More systematic training of parents with handing in video recordings for weekly review may facilitate faster acquisition of PRT fidelity [10]. Also, no information is available on the validity of current measures for PRT Fidelity of Implementation and future development of such measures should incorporate the use of PRT techniques during daily routines. Furthermore, the results of the study of Hardan et al. [10] indicated that Fidelity of Implementation of parents modified treatment effects for some, but not all, targeted verbal communication outcomes. Therefore, whether and how PRT Fidelity of Implementation modifies treatment effects is currently unclear and should be subject of future study.

Due to pragmatic reasons, only one PRT therapist (IvdB-S, shared first author) was trained to control the robot and use it within PRT, which may have induced bias. However, PRT training, intervision and supervision were similar for therapists in both PRT groups. Further studies should focus on training more therapists to control the robot, using software that is more easily understood by non-technically educated interventionists. Also, studies including the possibility of child’s (and parents’) choice in (PRT) intervention with and without the robot that systematically assesses reasons for choices, can provide insights in perspectives of children and parents on the usefulness of robot-assistance in interventions, for whom and why the robot is appealing to children with ASD. In this way, patient-tailored interventions can be improved. Furthermore, long-term follow-up measures should be included in future studies to assess whether PRT may generate long-lasting positive effects on both targeted child behavior and generalized outcomes.

In conclusion, this first RCT to PRT with and without robot-assistance including blinded raters suggests gains in general social communication in all intervention groups, with the largest gains in the robot-assisted PRT group. The following may be recommended for future research and clinical practice: (1) higher focus on child- and parent-related modifiers of intervention efficacy, within a higher sample size (2) better embedding of PRT in the school context, (3) better integration of PRT with co-interventions, (4) inclusion of long-term follow-up measures, (5) further development of adjustable game scenarios for robot-child interaction using software that is easily understood by non-technically trained therapists and (6) exploration of the mechanisms underlying treatment enhancing effects of robot-assistance. Together, these factors may contribute to further optimization of PRT for children with ASD.

## Supplementary Information

Below is the link to the electronic supplementary material.

Supplementary file1 (DOCX 20 kb)

Supplementary file2 (DOCX 14 kb)

Supplementary file3 (DOCX 23 kb)

## Data Availability

Data will be made available to investigators who provide a methodologically sound proposal for data use and achieving. Request can be sent by email to i.smeekens@karakter.com.
