# Supplementary material for: Pivotal Response Treatment with and without robot-assistance for children with autism: a randomized controlled trial
Source: Eur Child Adolesc Psychiatry. 2021 Jun 3;31(12):1871–83. doi: 10.1007/s00787-021-01804-8 (PMC9663375; doi:10.1007/s00787-021-01804-8)
Supplement: Supplementary file 1 — Supplementary file1 (DOCX 20 kb) [file 787_2021_1804_MOESM1_ESM.docx]

**Supplementary Information 1.** Description of game scenarios for robot-assisted PRT and implementation example

*Type of Robot and Controlling Robot Behavior*

In het robot-assisted PRT, a NAO robot was used in the first 15 minutes of all parent-child sessions. The NAO robot is a humanoid robot, developed by Aldebaran Robotics. The NAO robot is 58 cm in height and includes 25 degrees of freedom (i.e. motors to control the head, arms, hands and legs of the robot and LEDs to control the lights in the eyes and ears). With these joints, the robot can be programmed to perform human-like movements, such as cheering, shaking a hand, nodding and eye-blinking. Additionally, the speech of the NAO robot is produced by a speech synthesizer and played through integrated speakers. To control the robot, the visual programming environment TiViPe was used. In collaboration with the department of Industrial Design of the Eindhoven University of Technology, game scenarios were pre-programmed and a text-to-speech module was developed. These could be activated by the PRT therapist (by pressing a key on a laptop) when prompting or reinforcement was needed or when the pre-programmed scenario did not match the child's response in a specific situation.

*Game Scenarios for Robot-Child Interaction Development*

Game scenarios were created that included the following motivational techniques of PRT:

*1) Child choice and task variation:* Robot scenarios have been developed with nine different games that are developmentally appropriate for children aged 3-8 years and were often used during PRT for young children with ASD in the outpatient treatment facility of Karakter: three different puzzle games (i.e. a puzzle with different animals, a puzzle with a magnetic boat, a puzzle with trucks), three different Lego© games (building a plane, a car, or a house for older children; building a plane, ducks or a house with Duplo© for younger children), and three different card ("Quartet") games (cars with Frog & Friends, cards with sea animals, cards with fairytale characters). The type of game (i.e. puzzles, Lego©, or cards) could be selected by the therapist before start of each therapy session, based on parental information on child's preferences during the first therapy sessions and based on child's choice during therapy in later sessions. Additionally, the kind of game (i.e. type of either puzzle, or Lego©, or cards) was chosen by the child during the interaction with the robot and the therapist could select the robot scenario based on the choice of the child. Tasks were varied to maximize motivation. Also, the text-to-speech scenario could be activated when the child changed the subject of the robot-child conversation.

*2) Child attending and providing a clear opportunity to respond:* Learning opportunities were included in the game scenarios by 1) placing the desired materials in a closed box before the game starts, 2) providing the child with only parts of the game materials at once by using a box that the robot could slide open, and 3) providing only parts of information about a game. Learning opportunities were only provided if the child was interested in the robot and the game at that moment. If the child's attention was drawn to another game or subject, the therapist used the text-to-speech module to respond accordingly. If the child lost attention to the robot during the game, the game scenario was aborted to maintain child's motivation for the therapy session.

*3) Interspersing maintenance tasks:* For each child, a game scenario was selected that included learning opportunities for both maintained (easy) and new (difficult) tasks. Since target behavior and interests in game materials could differ highly between children with ASD, 9 different therapeutic game scenarios were created, each with 7 different levels of complexity. Also, the level of prompting (i.e. the help that the child received for showing appropriate behavior) could be adjusted throughout the game scenario and interspersed between easy (e.g. tell prompt) and difficult (e.g. wait prompt).

*4) Direct and natural reinforcement:* The game scenarios were designed to provide a direct and natural reinforcement upon the child's behavior. For instance, when the child asks: "robot, can you open de box?" the therapist directly controlled the robot in opening the box by pressing the appropriate key. Also, when a child takes an initiative that was not anticipated in the pre-programmed game scenario, the therapist could use the text-to-speech module to provide a direct and natural reinforcement.

*5) Reinforcement of attempts:* In the programming environment, the therapist pressed "y" (yes) within the game scenario when the child showed the target behavior or an appropriate attempt, and a direct and natural reinforcement was provided by the robot. However, when the child did not initiate spontaneously or the attempt was deemed inappropriate, the therapist pressed "n" (no) and the text-to-speech module was activated to prompt the child in showing an appropriate attempt to the target behavior.

*PRT techniques in robot-assisted implementation example*

While the child enters the treatment room, the robot is placed on the table and the child takes place before the robot. The therapist controls the robot in saying “hi” and offering a hand to the child. The child grabs the hand of the robot and the therapist controls the robot in shaking its hand and pull its hand back. Then, the robot places his arm on a box while saying: “there are some games in the box” (*Child attending and providing a clear opportunity to respond*). If the child does not initiate spontaneously, the therapist can controls the robot in providing prompts (i.e. waiting prompt: waiting for 7 seconds; open question prompt: “what can you ask me now?”; fill in prompt: you can ask me: “can you…” or tell prompt: you can ask: “can you remove your arm?”). The child can access three boxes of Lego©, that are placed in the larger box after providing an appropriate initiation. The child can choose one of the boxes (*Child choice and task variation*) and the therapist removes the other boxes from the table while placing a sliding box in front of the robot. The child starts with building his/her preferred Lego© and notices that not all the required blocks are in the box. The child asks the robot: “can I have more blocks?” and the therapist controls the robot in opening the sliding box with its hand (*Direct and natural reinforcement)*. The child continues building with the Lego and two more learning opportunities are provided on asking more blocks. When the child is almost finished with building a Lego house, the therapist controls the robot in saying: “you make a nice car” while blinking with its eyes. This is a learning opportunity for protesting (*Interspersing maintenance tasks with new tasks)* and when the child does not initiate spontaneously, the therapist controls the robot in providing a tell prompt: you can say: “this is not a car”. When the child uses an adequate protesting attempt towards the robot, the therapist controls the robot in saying: “Haha, I have made a joke. Of course it is a house!” (*Rewarding attempts*). And then the child continues building the house. When the house is finished, the therapist controls the robot in saying: “I know a sound that you can hear in a house”. When the child initiates by asking the robot to let him/her hear the sound, the therapist controls the robot in making the sound of a doorbell. After a few more learning opportunities for asking for an object/activity (maintenance task) and protesting (new task), the robot-child interaction is finished by providing giving the robot a hand, saying goodbye and turning the robot off. Then, the PRT session is continued with parent-child interaction and coaching by the therapist.
