# Supplementary material for: Pivotal Response Treatment with and without robot-assistance for children with autism: a randomized controlled trial
Source: Eur Child Adolesc Psychiatry. 2021 Jun 3;31(12):1871–83. doi: 10.1007/s00787-021-01804-8 (PMC9663375; doi:10.1007/s00787-021-01804-8)
Supplement: Supplementary file 2 — Supplementary file2 (DOCX 14 kb) [file 787_2021_1804_MOESM2_ESM.docx]

**Supplementary Information 2.** Sensitivity analyses on parent-rated SRS

Sensitivity analyses for 3-group comparisons on percentages of clinical responders on the parent-rated SRS (see Table 3).

Table 3.

*Sensitivity analyses on SRS percentages of clinical responders*

|  |  |  | | | | |  | | | | Chi-square analyses results | | | |
| --- | --- | --- | --- | --- | --- | --- | --- | --- | --- | --- | --- | --- | --- | --- |
|  |  | % Endpoint | | | | | % Follow-up | | | | Endpoint | | Follow-up | |
|  |  | PRT | PRT+  robot | | TAU | PRT | | PRT+  robot | | TAU | *Х*²(df) | *p* | *Х*²(df) | *p* |
| Cut-off |  |  | | | | | | | | | | | | |
| **≥ 25% decrease** |  | **13.0** | **28.0** | **18.2** | | **12.0** | **50.0** | | **20.0** | | **1.72 (2)** | **.419** | **9.66 (2)** | **.008**** |
| ≥ 20% decrease |  | 24.0 | 36.0 | 30.4 | | 24.0 | 52.0 | | 21.7 | | 0.86 (2) | .652 | 6.33 (2) | .042* |
| ≥ 30% decrease |  | 4.0 | 24.0 | 8.7 | | 12.0 | 40.0 | | 4.3 | | 5.04 (2) | .081 | 11.09 (2) | .004** |
| ≥ 35% decrease |  | 4.0 | 12.0 | 0.0 | | 12.0 | 36.0 | | 4.3 | | 3.49 (2) | .175 | 9.08 (2) | .011** |
| ≥ 40% decrease |  | 4.0 | 8.0 | 0.0 | | 8.0 | 32.0 | | 4.3 | | 1.95 (2) | .378 | 8.64 (2) | .013** |

*Note:* ^*^ *p* < .05, ^**^ *p* < .01

df = degrees of freedom; p = p-value (two-tailed); PRT = group of participants who received Pivotal Response Treatment; PRT+robot = group of participants who received robot-assisted Pivotal Response Treatment; SRS = Social Responsiveness Scale; TAU = group of participants who received treatment-as-usual; *Х*² = test statistic resulting from Chi squared analyses, 3-group = comparison between PRT, PRT+robot and TAU.
