# Supplementary material for: Pivotal Response Treatment with and without robot-assistance for children with autism: a randomized controlled trial
Source: Eur Child Adolesc Psychiatry. 2021 Jun 3;31(12):1871–83. doi: 10.1007/s00787-021-01804-8 (PMC9663375; doi:10.1007/s00787-021-01804-8)
Supplement: Supplementary file 3 — Supplementary file3 (DOCX 23 kb) [file 787_2021_1804_MOESM3_ESM.docx]

**Supplementary Information 3.** Results of exploratory within-group analyses

In Table 4, means and standard deviations (SD) per group and results of exploratory paired-sample t-tests for within-group effects are shown.

*SRS*

All three groups showed lower SRS total scores, indicating higher generalized social-communicative skills rated by parents from baseline to endpoint (*p’s* = .000 to .047). Significant improvements were also maintained at follow-up, except for the PRT group in which change from baseline showed a marginal trend towards significance (*p*= .053). At both endpoint and follow-up, large effect sizes were found for the PRT+robot group (*d* = 0.75 and 0.80 respectively) and small effect sizes were found for the PRT group (*d* = 0.34 and 0.23 respectively) and TAU group (*d* = 0.30 and 0.23 respectively). On the SRS rated by teachers, no significant differences were found between baseline and endpoint and between baseline and follow-up for any of the groups (all *p* > .05).

*ADOS-2 by blinded rater*

Exploratory paired-sample t-tests for indicated no significant changes in ADOS-2 total scores or calibrated severity scores (CSS) from baseline to endpoint in any of the groups.

*OBVL*

Exploratory paired-sample t-tests showed no significant change from baseline to endpoint in any of the groups. However, the PRT+robot group and TAU group had a lower OBVL score indicating lower parenting stress at follow-up compared with baseline while the PRT group showed no significant difference.

*Spontaneous self-initiations.*

Wilcoxon signed ranks test for related samples indicated that both PRT groups showed an increase in the percentage spontaneous self-initiations during a semi-structured therapist-child interaction at endpoint and follow-up compared with baseline.

| Table 4. | | |  |  |  |  |  |  |  |  |  |
| --- | --- | --- | --- | --- | --- | --- | --- | --- | --- | --- | --- |
| *Means and standard deviations per group results of within-group analyses.* | | | | | |  |  |  |  |  |  |
|  | PRT (*n* = 25) | | | |  |  |  |  |  |  |  |
|  | Baseline | Week 10 | Endpoint | Follow-up | Baseline to endpoint | | |  | Baseline to follow-up | | |
|  | M (SD) | M (SD) | M (SD) | M (SD) | *t* | *p* | *d* |  | *t* | *p* | *d* |
| SRS |  |  |  |  |  |  |  |  |  |  |  |
| parents – total score | 84.47 (22.03) | 83.63 (23.29) | 77.79 (22.81) | 78.63 (26.63) | 2.97 | **.007**** | 0.34 |  | 2.97 | .053 | 0.23 |
| teachers – total score | 83.23 (25.90) | 81.42 (24.35) | 79.86 (24.17) | 75.71 (23.34) | 0.58 | .567 | 0.08 |  | 1.36 | .188 | 0.24 |
| CGI-I |  |  |  |  |  |  |  |  |  |  |  |
| score | - | 3.32 (1.25) | 3.04 (1.40) | 2.21(1.06) |  |  |  |  |  |  |  |
| ADOS-2 |  |  |  |  |  |  |  |  |  |  |  |
| total score | 10.05 (3.42) | - | 9.77(3.44) | - | 0.54 | .593 | 0.08 |  |  |  |  |
| CSS | 5.91 (2.18) | - | 5.91 (1.82) | - | 0.00 | 1.000 | 0.00 |  |  |  |  |
| OBVL |  |  |  |  |  |  |  |  |  |  |  |
| total score | 60.40 (13.70) | 61.77 (14.54) | 59.92 (12.94) | 59.40 (12.95) | 1.04 | .311 | 0.11 |  | 0.51 | .612 | 0.08 |
| Self-initiations |  |  |  |  |  |  |  |  |  |  |  |
| % spontenous | 38.97 (25.05) | 63.43 (24.22) | 68.74 (25.94) | 76.67 (15.45) | -2.84^a^ | **.004**** |  |  | -3.12^a^ | **.002**** |  |
|  | PRT + robot (*n* = 25) | | | |  |  |  |  |  |  |  |
|  | Baseline | Week 10 | Endpoint | Follow-up | Baseline to Endpoint | | |  | Baseline to follow-up | | |
|  | M (SD) | M (SD) | M (SD) | M (SD) | *t* | *p* | *d* |  | *t* | *p* | *d* |
| SRS |  |  |  |  |  |  |  |  |  |  |  |
| parents – total score | 84.19 (22.56) | 79.84 (24.08) | 68.16 (20.31) | 63.38 (25.84) | 5.22 | **<.001***** | 0.75 |  | 5.27 | **<.001***** | 0.84 |
| teachers – total score | 76.72 (24.26) | 69.00 (24.59) | 65.78 (20.66) | 70.87 (26.69) | 2.05 | .054 | 0.45 |  | 1.37 | .189 | 0.32 |
| CGI-I |  |  |  |  |  |  |  |  |  |  |  |
| score | - | 2.40 (1.10) | 2.33 (0.76) | 2.21 (1.06) |  |  |  |  |  |  |  |
| ADOS-2 |  |  |  |  |  |  |  |  |  |  |  |
| total score | 10.00 (2.45) | - | 8.59 (3.38) | - | 1.97 | .063 | 0.48 |  |  |  |  |
| CSS | 5.95 (1.43) | - | 5.18 (2.06) | - | 1.60 | .125 | 0.43 |  |  |  |  |
| OBVL |  |  |  |  |  |  |  |  |  |  |  |
| total score | 53.56 (12.74) | 51.72 (12.10) | 50.32 (11.32) | 48.86 (11.69) | 1.71 | .101 | 0.27 |  | 2.09 | **.048*** | 0.38 |
| Self-initiations |  |  |  |  |  |  |  |  |  |  |  |
| % spontenous | 44.31 (20.82) | 66.09 (17.71) | 74.86 (14.89) | 71.59 (13.98) | -3.88^a^ | **<.001***** |  |  | -3.72^a^ | **<.001***** |  |
|  | TAU (*n* = 23) | | | |  |  |  |  |  |  |  |
|  | Baseline | Week 10 | Endpoint | Follow-up | Baseline to Endpoint | | |  | Baseline to follow-up | | |
|  | M (SD) | M (SD) | M (SD) | M (SD) | *t* | *p* | *d* |  | *t* | *p* | *d* |
| SRS |  |  |  |  |  |  |  |  |  |  |  |
| parents – total score | 85.51 (26.47) | 81.03 (26.25) | 77.33 (24.80) | 79.70 (31.65) | 2.24 | **.036*** | 0.30 |  | 2.58 | **.018*** | 0.23 |
| teachers – total score | 86.69 (25.02) | 78.53 (19.31) | 77.63 (26.25) | 76.93 (24.17) | 1.43 | .167 | 0.36 |  | 1.75 | .094 | 0.40 |
| CGI-I |  |  |  |  |  |  |  |  |  |  |  |
| score | - | 3.10 (1.09) | 2.67 (1.07) | 3.10 (1.09) |  |  |  |  |  |  |  |
| ADOS-2 |  |  |  |  |  |  |  |  |  |  |  |
| total score | 12.17 (5.09) | - | 10.94 (4.78) | - | 1.76 | .096 | 0.25 |  |  |  |  |
| CSS | 6.39 (1.98) | - | 6.11(2.30) | - | .70 | .491 | 0.13 |  |  |  |  |
| OBVL |  |  |  |  |  |  |  |  |  |  |  |
| total score | 61.91 (16.23) | 59.66 (17.73) | 57.90 (17.38) | 56.76 (16.78) | 1.62 | .121 | 0.16 |  | 2.17 | **.043*** | 0.24 |

*Note:* ^*^ *p* < .05, ^**^ *p* < .01, ^***^ *p* < .001, ^a^ represents z-statistic resulting from Wilcoxon signed ranks test

ADOS-2 = Autism Diagnostic Observation Schedule second edition; CGI-I = Clinical Global Impression – Improvement scale; CSS = calibrated severity score; *d* = effect size; M = mean; n = number of participants; OBVL = Dutch Opvoedingsbelasting vragenlijst; PRT = group of participants who received Pivotal Response Treatment; *p* = *p*-value (two-tailed); PRT+robot = group of participants who received robot-assisted Pivotal Response Treatment; RCI = reliable clinical improvement; SD = standard deviation; SRS = Social Responsiveness Scale; *t* = test statistic resulting from paired-sample t-tests; TAU = group of participants who received treatment-as-usual.
